# Stochastic Proximal Gradient Algorithms for Multi-Source Quantitative Photoacoustic Tomography

**DOI:** 10.3390/e20020121

**Published:** 2018-02-11

**Authors:** Simon Rabanser, Lukas Neumann, Markus Haltmeier

**Affiliations:** 1 Department of Mathematics, University of Innsbruck, Technikerstraße 13, A-6020 Innsbruck, Austria; 2 Institute of Basic Sciences in Engineering Science, University of Innsbruck, Technikerstraße 13, A-6020 Innsbruck, Austria

**Keywords:** photoacoustic tomography, image reconstruction, radiative transfer equation, multilinear inverse problem, limited view, stochastic gradient method, limited data, Dykstra algorithm

## Abstract

The development of accurate and efficient image reconstruction algorithms is a central aspect of quantitative photoacoustic tomography (QPAT). In this paper, we address this issues for multi-source QPAT using the radiative transfer equation (RTE) as accurate model for light transport. The tissue parameters are jointly reconstructed from the acoustical data measured for each of the applied sources. We develop stochastic proximal gradient methods for multi-source QPAT, which are more efficient than standard proximal gradient methods in which a single iterative update has complexity proportional to the number applies sources. Additionally, we introduce a completely new formulation of QPAT as multilinear (MULL) inverse problem which avoids explicitly solving the RTE. The MULL formulation of QPAT is again addressed with stochastic proximal gradient methods. Numerical results for both approaches are presented. Besides the introduction of stochastic proximal gradient algorithms to QPAT, we consider the new MULL formulation of QPAT as main contribution of this paper.

## 1. Introduction

**Figure 1:**
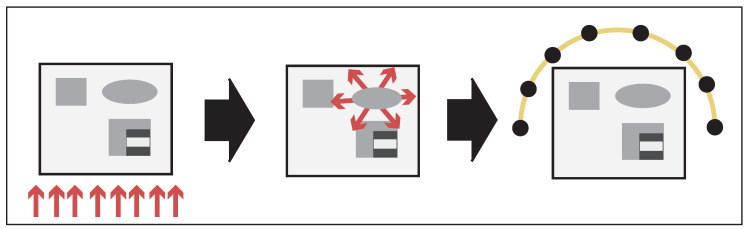
Basic principles of PAT. **Left**: the investigated object is illuminated with a short optical pulse; **Middle**: due to the thermoelastic effect, the absorbed light distribution induces an acoustic pressure wave depending on internal tissue properties; **Right**: the acoustic pressure wave is measured outside the object and used to reconstruct an image of the interior.

Photoacoustic tomography (PAT) is an emerging imaging modality, which combines the benefits of pure ultrasound imaging (high resolution) with those of pure optical tomography (high contrast); see [1,2]. The basic principle of PAT is as follows (see Figure 1): A semitransparent sample such as a part of a human patient is illuminated with short pulses of optical radiation. A fraction of the optical energy is absorbed inside the sample, which causes thermal heating, expansion, and a subsequent acoustic pressure wave depending on the interior absorbing structure of the sample. The acoustic pressure is measured outside of the sample and used to reconstruct an image of the interior.

One important reconstruction problem in PAT is recovering the initial pressure distribution (see, for example, [3,4,5,6,7,8,9,10]). The initial pressure distribution only provides qualitative information about the tissue-relevant parameters, as it is the product of the optical absorption coefficient and the spatially varying optical intensity, which again indirectly depends on the tissue parameters. Quantitative photoacoustic tomography (QPAT) addresses this issue and aims at quantitatively estimating the tissue parameters by supplementing the inversion of the acoustic wave equation with an inverse problem for light propagation (see, for example, [11,12,13,14,15,16,17,18,19,20,21,22,23,24,25,26]).

### 1.1. Multi-Source QPAT

In this paper, we consider image reconstruction in QPAT using multiple sources. We allow limited view measurements, where, for each illumination, partial data are collected only from a certain angular domain. For modeling the light transport, we use the radiative transfer equation (RTE), which is commonly considered as a very accurate model for light transport in tissue (see, for example, [27,28,29,30]). In particular, opposed to the diffusion approximation, the RTE allows for modeling directed optical radiation, which is required for a reasonable QPAT forward model. Additionally, it allows for including internal voids as regions of low scattering. As proposed in [18], we work with a single-stage reconstruction procedure for QPAT, where the optical parameters are reconstructed directly from the measured acoustical data. The image reconstruction problem of multi-source QPAT using *N* different sources can be formulated as a system of nonlinear equations (see, for example, [18,31])

(1) $$F_{i}\left(\mu \right)=v_{i}\;\;\mathrm{for}\;i=1,\ldots ,N\;.$$

Here, $F_{i}$ is the operator that maps the unknown parameter pair $\mu =(\mu_{a},\mu_{s})$ consisting of the absorption coefficient $\mu_{a}:\Omega \to R$ and the scattering coefficient $\mu_{s}:\Omega \to R$ to the measured acoustic data $v_{i}$ corresponding to the *i*-th source distribution (see Section 2 for precise definitions). There are two main classes of methods for solving the nonlinear inverse problem (1), namely, Tikhonov type regularization on the one and iterative regularization methods on the other hand [32,33,34]. Both approaches are based on rewriting (1) as a single equation F(μ)=v with forward operator $F={\left(F_{i}\right)}_{i=1}^{N}$ and data $v={\left(v_{i}\right)}_{i=1}^{N}$. In Tikhonov regularization, one defines approximate solutions as minimizers of the penalized least squares functional $\frac{1}{2}{∥F(\mu )-v∥}^{2}+\lambda R\left(\mu \right)$. Here, R(·) is an appropriate regularization functional included to stabilize the inversion process and λ a regularization parameter that has to be carefully chosen depending on the data and the noise. In iterative regularization methods, stabilization is achieved via early stopping of iterative schemes. In such a situation, one usually applies iterative optimization techniques designed for minimizing the un-regularized least squares functional $\frac{1}{2}{∥F(\mu )-v∥}^{2}$, and the iteration index plays the role of the regularization parameter.

Tikhonov type as well as iterative regularization methods can both be formulated as finding a solution of the optimization problem

(2) $$\left\{\begin{array}{cc} \min & \frac{1}{2}\sum_{i=1}^{N}{∥F_{i}\left(\mu \right)-v_{i}∥}^{2}+G\left(\mu \right),\\ \mathrm{with}\; & \mu \in L^{2}\left(\Omega \right)\times L^{2}\left(\Omega \right)\;. \end{array}\right.$$

In iterative regularization methods, one takes $G=\chi_{D}$, the characteristic function of the domain of definition D of the forward operator (taking the value 0 in and the value ∞ outside of D). In Tikhonov regularization, we take $G=\chi_{D}+\lambda R$. Well established algorithms for solving Equation (2) are proximal gradient algorithms [35,36], which can be written in the form

(3) $$\mu_{k+1}={\mathrm{prox}}_{s_{k}G}\left(\mu_{k}-s_{k}\sum_{i=1}^{N}F_{i}^{\prime }{\left(\mu_{k}\right)}^{*}\left(F_{i}\left(\mu_{k}\right)-v_{i}\right)\right)\;.$$

Here, ${\mathrm{prox}}_{s_{k}G}$ is the proximity operator and $s_{k}$ the positive step size; $F_{i}^{\prime }\left(\mu_{k}\right)$ denotes the derivative of the *i*-th forward operator evaluated at $\mu_{k}$ with $F_{i}^{\prime }{\left(\mu_{k}\right)}^{*}$ being its Hilbert space adjoint.

### 1.2. Stochastic Proximal Gradient Algorithms

Each iteration in the proximal gradient algorithm (3) can be numerically quite expensive, since it requires solving the forward and adjoint problems for all *N* equations in (1). In many cases, stochastic (proximal) gradient methods turn out to be more efficient since these methods only consider one of the equations in (1) per iteration. The stochastic proximal gradient method (see, for example, [37,38,39,40,41,42] and the references therein) for solving (2) is defined by

$$\mu_{k+1}={\mathrm{prox}}_{s_{k}G}\left(\mu_{k}-s_{k}F_{i(k)}^{\prime }{\left(\mu_{k}\right)}^{*}\left(F_{i}\left(\mu_{i(k)}\right)-v_{i}\right)\right)\;,$$

where $i\left(k\right)\in \left\{1,\ldots ,N\right\}$ corresponds to one of the equations in (1) that is selected randomly for the update in the *k*-th iteration. In opposition to the standard proximal gradient method, this requires solving only one forward and one adjoint problem per iteration. Therefore, one iterative step is much cheaper for the stochastic gradient method than for the full gradient method. In the case of no regularization, λ=0, the stochastic proximal gradient method reduces to the Kaczmarz method for inverse problems studied in [43,44,45].

The computationally most expensive task in the above methods is the numerical solution of the RTE. In this paper, we therefore additionally study a reformulation of the inverse problem of QPAT avoiding the computation of a solution of the RTE. For this purpose, the inverse problem is reformulated as multilinear inverse problem (35), where the RTE is added as a constraint instead of explicitly including its solution. The new formulation will be again addressed by Tikhonov regularization in combination with proximal stochastic gradient methods as discussed in Section 4.

Note that, in QPAT, it has often been assumed that the initial pressure distribution (corresponding to each illumination) is already recovered from acoustic measurements (see, for example, [13,16,23,25,26,46,47,48,49]). Research was focused on inverting the light propagation in tissues either modeled by the RTE or the diffusion approximation. In the case that acoustic measurements are only known on parts of the boundary, reconstruction of the initial pressure distribution is not possible in a stable manner. In order to obtain stable reconstruction results in [18], we propose a single-stage approach for QPAT, where the optical parameters are directly recovered from the acoustic boundary data. Throughout this paper, we will make use of this approach, which delivers stable results especially in the limited view situation. In opposition to [18], in this paper, we introduce (proximal) stochastic gradient methods, which effectively exploit the multi-illumination structure and turn out to be faster than the standard proximal gradient methods.

### 1.3. Outline

The remainder of this paper is organized as follows. In Section 2, we provide the mathematical model for QPAT (the forward problem) using the RTE. We allow multiple sources and partial acoustic measurements. We also recall known results for QPAT including differentiability of the forward problem. In Section 3, we address the inverse problem of QPAT using Tikhonov regularization and study the proximal stochastic gradient method for its solution. The new reformulation of the inverse problem of QPAT as a multilinear inverse problem is presented in Section 4. For the solution of the proposed formulation, we again develop proximal gradient methods. Numerical results are presented in Section 5. The paper is concluded with a summary and outlook presented in Section 6.

## 2. The Forward Problem in QPAT

The image reconstruction problem of QPAT can be written as the system (1) of nonlinear equations, where the forward operators $F_{i}$ map tissue relevant parameters to acoustic data sets recorded in specific regions outside the tissue. Precise formulations will be given in this section.

### 2.1. Mathematical Notation

We fix some mathematical notation that is used throughout this paper. We denote by $\Omega \subseteq R^{d}$ a convex domain with piecewise smooth boundary modeling our domain of interest, with $d\in \left\{2,3\right\}$ denoting the spatial dimension. In order to be able to impose appropriate boundary conditions for the RTE, it is convenient to split the set $\Gamma :=\partial \Omega \times S^{d-1}$ into inflow and outflow boundaries,

$$\begin{array}{cc} \Gamma_{-} & :=\left\{\left(x,\theta \right)\in \partial \Omega \times S^{d-1}|\nu \left(x\right)\;\cdot \;\theta \leq 0\right\}\;,\\ \Gamma_{+} & :=\left\{\left(x,\theta \right)\in \partial \Omega \times S^{d-1}|\nu \left(x\right)\;\cdot \;\theta >0\right\}\;, \end{array}$$

with ν(x) denoting the outward pointing unit normal at x∈∂Ω and x·y the standard inner product in $R^{d}$. We write $B_{R}=\left\{x\in R^{n}|∥x∥<R\right\}$ for the ball of radius *R* centered at the origin and suppose $B_{R}⊇\Omega$.

By $L^{2}\left(\Omega \right)$ and $L^{2}\left(\Omega \times S^{d-1}\right)$, we denote the Hilbert spaces of square integrable functions on Ω and $\Omega \times S^{d-1}$, respectively. By $L^{2}\left(\Gamma_{-},\left|\nu \;\cdot \;\theta \right|\right)$, we denote the space of all $q_{o}:\Gamma_{-}\to R$ for which ${∥q_{o}∥}_{L^{2}\left(\Gamma_{-},\left|\nu \;\cdot \;\theta \right|\right)}^{2}:=\int_{\Gamma_{-}}{\left|q_{o}\left(x,\theta \right)\right|}^{2}\left|\nu \;\cdot \;\theta \right|d\left(x,\theta \right)$ is finite. We further write

$$\begin{array}{cc} {∥\Phi ∥}_{W}^{2} & :={∥\Phi ∥}_{L^{2}\left(\Omega \times S^{d-1}\right)}^{2}+∥\theta \;\cdot \;\nabla_{x}{\Phi ∥}_{L^{2}\left(\Omega \times S^{d-1}\right)}^{2}+{∥\Phi |}_{\Gamma_{-}}{∥}_{L^{2}\left(\Gamma_{-},\left|\nu \;\cdot \;\theta \right|\right)}^{2}\;,\\ {∥v∥}_{Y}^{2} & :=\int_{0}^{\infty }\int_{\partial B_{R}}{\left|v(x,t)\right|}^{2}t\;dxdt\;, \end{array}$$

and define

$$\begin{array}{cc} Q & :=L^{2}\left(\Omega \times S^{d-1}\right)\times L^{2}\left(\Gamma_{-},\left|\nu \;\cdot \;\theta \right|\right),\\ X & :=L^{2}\left(\Omega \right)\times L^{2}\left(\Omega \right),\\ W & :=\{\Phi :\Omega \times S^{d-1}\to R|{∥\Phi ∥}_{W}<\infty \},\\ Y & :=\{v:\partial B_{R}\times \left(0,\infty \right)\to R|{∥v∥}_{Y}<\infty \}\;. \end{array}$$

The inner products in Q, X, W , Y will be denoted by ${\langle \;\;\cdot \;\;,\;\;\cdot \;\;\rangle }_{Q}$, ${\langle \;\;\cdot \;\;,\;\;\cdot \;\;\rangle }_{X}$, ${\langle \;\;\cdot \;\;,\;\;\cdot \;\;\rangle }_{W}$, ${\langle \;\;\cdot \;\;,\;\;\cdot \;\;\rangle }_{Y}$, respectively. The subspace of all Φ∈W with ${\Phi |}_{\Gamma_{-}}=0$ will be denoted by $W_{0}$.

Elements in X will be written in the form $\mu =(\mu_{a},\mu_{s})$ and are the parameters we aim to determine. They are actually required to be contained in the convex subset

(4) $$D\left(T\right):=\left\{\mu \in X|0\leq \mu_{a}\leq {\bar{\mu }}_{a}\;,0\leq \mu_{s}\leq {\bar{\mu }}_{s}\right\}\;,$$

where ${\bar{\mu }}_{a},{\bar{\mu }}_{s}>0$. Elements in Q will be written in the form $q=(q_{o},q_{i})$ and model the optical sources. Elements in W describe the optical radiation, and elements in Y the measured acoustic data.

### 2.2. The Radiative Transfer Equation

To specify the forward operators, we require mathematical models for the light propagation, the conversion of optical into acoustic energy, and the propagation of the acoustic waves. These models will be presented in the rest of this section.

We model the optical radiation by a function $\Phi :\Omega \times S^{d-1}\to R$, where Φ(x,θ) is the density of photons at position x∈Ω and propagating into direction $\theta \in S^{d-1}$. The interaction of the photons with the background are described by absorption coefficient $\mu_{a}:\Omega \to R$, the scattering coefficient $\mu_{s}:\Omega \to R$, and the scattering operator K:Φ↦KΦ, taking the form (see [27,30])

(5) $$\forall \left(x,\theta \right)\in \Omega \times S^{d-1}:\;K\Phi \left(x,\theta \right)=\int_{S^{d-1}}k\left(\theta ,\theta^{\prime }\right)\Phi \left(x,\theta^{\prime }\right)d\theta^{\prime }\;,$$

with scattering kernel $k:S^{d-1}\times S^{d-1}\to R$. The absorption coefficient describes the ability of the background to absorb photons and the scattering coefficient describes the amount of photon scattering. The scattering kernel $k\left(\theta ,\theta^{\prime }\right)$ describes the redistribution of velocity directions due to interaction of the photons with the background. From physical considerations, it is natural to assume *k* to be measurable, symmetric, nonnegative, and to satisfy $\int_{S^{d-1}}k\left(\;\;\cdot \;\;,\theta^{\prime }\right)d\theta^{\prime }=1$. In this article, we are concerned with the situation when the kernel is known a priori.

The photon density Φ(x,θ) is supposed to satisfy the stationary radiative transfer equation (RTE),

(6) $$\left(\theta \;\cdot \;\nabla_{x}+\mu_{a}+\mu_{s}\left(I-K\right)\right)\Phi \left(x,\theta \right)=q_{i}\left(x,\theta \right)\;\;\mathrm{for}\;\left(x,\theta \right)\in \Omega \times S^{d-1}$$

with boundary conditions

(7) $${\Phi |}_{\Gamma_{-}}\left(x,\theta \right)=q_{o}\left(x,\theta \right)\;\;\mathrm{for}\;\left(x,\theta \right)\in \Gamma_{-}\;.$$

Here, $q_{i}:\Omega \times S^{d-1}\to R$ denotes an internal photon source and $q_{o}:\Gamma_{-}\to R$ a prescribed boundary source pattern. Note that PAT uses very short light pulses (below microseconds) and that light propagation happens on time scales much shorter than the scale of acoustic wave propagation. This justifies the use of the stationary case for the RTE; see [12] for a more complete discussion.

**Theorem** **1** (Well-posedness of the RTE). *For every μ∈D(T) and q∈Q, the stationary RTE (6) admits a unique solution Φ∈W. Moreover, there exists a constant C only depending on the parameters ${\bar{\mu }}_{a},{\bar{\mu }}_{s}>0$ (defining the domain D(T)), such that*

(8) $${∥\Phi ∥}_{W}\leq C\;\left({∥q_{i}∥}_{L^{2}}+{∥q_{o}∥}_{L^{2}}\right)\;.$$

**Proof.**  See [50]. ☐

**Definition** **1** (Solution operator for the RTE). *The solution operator for the RTE is defined by*

$$T:Q\times D\left(T\right)\to W:\left(q,\mu \right)\mapsto T\left(q,\mu \right):=\Phi \;,$$

*where* Φ *denotes the unique solution of (6).*

Theorem 1 guarantees that the operator T, mapping (q,μ)∈Q×D(T) to the solution of the RTE, is well defined. Note that in the actual application $q=(q_{i},q_{o})\in Q$ are prescribed sources, and $\mu =\left(\mu_{a},\mu_{s}\right)\in D\left(T\right)$ are the unknown parameters to be recovered.

### 2.3. Heating Operator

Due to the spatially varying absorption of photons, the tissue is locally heated and emits an acoustic pressure wave. The acoustic source is proportional to the amount of absorbed photons, the light intensity and the so-called Grüneisen parameter γ describing the efficiency of conversion of optical to acoustical energy. We assume γ to be constant and after appropriate re-scaling we take γ=1; for more details about the Grüneisen parameter, we refer to [17]. Therefore, the conversion of the optical energy into acoustic pressure wave is described by the heating operator defined as follows.

**Definition** **2** (Heating operator). *The heating operator is defined by*

(9) $$\begin{array}{cc} H:Q\times D(T) & \to L^{2}\left(\Omega \right)\\ \left(q,\mu \right) & \mapsto \mu_{a}\int_{S^{d-1}}T\left(q,\mu \right)\left(\;\;\cdot \;\;,\theta \right)d\theta \;. \end{array}$$

If one introduces the averaging operator $A:W\mapsto L^{2}\left(\Omega \right)$ defined by $A\Phi =\int_{S^{d-1}}\Phi \left(\;\;\cdot \;\;,\theta \right)d\theta$ one may write the heating operator in the form

$$H\left(q,\mu \right)=\mu_{a}\;A\circ T\left(q,\mu \right)\;\;\mathrm{for}\;\left(q,\mu \right)\in Q\times D\left(T\right)\;.$$

Because T(q,μ) models the photon density, A∘T(q,μ) actually models the total light intensity. The heating operator is therefore given by the product of the absorption coefficient and the light intensity. The averaging operator A is well defined and bounded and therefore the heating operator is well defined as a mapping between Q×D(T) and $L^{2}\left(\Omega \right)$.

### 2.4. The Wave Equation

The local heating causes an acoustic pressure wave, where the initial pressure distribution $p_{0}$ is proportional to a fraction of the absorbed energy. Assuming constant speed of sound and after rescaling, the induced acoustic pressure $p:R^{d}\times \left(0,\infty \right)\to R$ satisfies the free-space wave equation:

(10) $$\left\{\begin{array}{cc} \left(\partial_{t}^{2}-\Delta \right)p\left(x,t\right)=0 & \mathrm{for}\;\left(x,t\right)\in R^{d}\times \left(0,\infty \right),\\ p\left(x,0\right)=p_{0}\left(x\right) & \mathrm{for}\;x\in R^{d},\\ \partial_{t}p\left(x,0\right)=0 & \mathrm{for}\;x\in R^{d}\;. \end{array}\right.$$

Here, the function $p_{0}$ vanishes outside $B_{R}$, the ball of radius *R*, and acoustic data are collected on a subset of $\partial B_{R}\times \left(0,\infty \right)$ that we denote by Λ×(0,∞). Recall that coupling of the RTE and the wave equation happens in such a way that the result of the heating operator H(q,μ) acts as initial sound source $p_{0}$ depending on tissue parameters; see Definition 4. Standard existence and uniqueness theory for hyperbolic equations guarantees that, for any $p_{0}\in H^{1}$, (10) has a unique solution $p\in H^{1}$, which continuously depends on $p_{0}$. Taking the trace results in loss of regularity by degree 1/2. Therefore, $p_{0}{\mapsto p|}_{\partial B_{R}\times \left(0,\infty \right)}$ is continuous between $H^{1}$ and $H^{1/2}$. The following Lemma implies the much stronger result that $p_{0}{\mapsto p|}_{\partial B_{R}\times \left(0,\infty \right)}$ is actually an $L^{2}$-isometry.

**Lemma** **1.** *Let $p_{0}\in C^{\infty }\left(R^{n}\right)$ have support in $B_{R}$ and let p denote the solution of (10). Then,*

(11) $$\int_{B_{R}}\int_{0}^{\infty }{\left|p(x,t)\right|}^{2}tdt\;dx=\frac{R}{2}\int_{B_{R}}{\left|p_{0}\left(x\right)\right|}^{2}dx\;.$$

**Proof.**  See [51] for *d* odd and [52] for *d* even. ☐

**Definition** **3.** *We define the solution operator with full boundary data for the wave Equation (10) by*

(12) $$U:C^{\infty }\left(B_{R}\right)\subseteq L^{2}\left(B_{R}\right)\to Y:p_{0}{\mapsto p|}_{\partial B_{R}\times \left(0,\infty \right)}\;,$$

*where p denotes the solution of* (10).

According to Lemma 1, the operator U can be uniquely extended to a bounded linear operator defined on $L^{2}\left(B\right)$, denoted again by $U:L^{2}\left(B_{R}\right)\to Y$. The partial acoustic measurements made on $\Lambda \subseteq \partial B_{R}$ are then modeled by $\chi_{\Lambda \times (0,\infty )}Up_{0}$.

### 2.5. Analysis of the Forward Problem in Multi-Source QPAT

We assume that we perform *N* individual experiments, where each experiment consists of separate optical sources and separate acoustic measurements. For the *i*-th experiment, we denote the source term by $q_{i}\in Q$ and assume the acoustic measurements are made on $\Lambda_{i}\times \left(0,T_{i}\right)\subseteq \partial B_{R}\times \left(0,\infty \right)$.

**Definition** **4.** *For any i∈{1,…,N}, we denote*

$$\begin{array}{cc} T_{i}: & D\left(T\right)\to W:\mu \mapsto T\left(q_{i},\mu \right),\\ H_{i}: & D\left(T\right)\to L^{2}\left(\Omega \right):\mu \mapsto \mu_{a}\left(A\circ T_{i}\right)\left(\mu \right),\\ U_{i}: & L^{2}\left(\Omega \right)\to Y:p_{0}\mapsto \chi_{\Lambda_{i}\times \left(0,T_{i}\right)}Up_{0},\\ F_{i}: & D\left(T\right)\to Y:\mu \mapsto \left(U_{i}\circ H_{i}\right)\left(\mu \right)\;. \end{array}$$

*Here, $T_{i}$ denotes the i-th solution operator for the RTE, $H_{i}$ the i-th heating operator, $U_{i}$ the i-th partial solution operator for the wave equation, and $F_{i}$ the i-th forward operator.*

Recall that T stands for the solution operator for the RTE (6) given in Definition 1, $A\Phi =\int_{S^{d-1}}\Phi \left(\;\;\cdot \;\;,\theta \right)d\theta$ is the averaging operator, and U the solution operator for the wave Equation (10); see Definition 3. The operator $T_{i}$ models the photon transport and its solution (via the heating operator) acts as input for the solution of the wave equation and thereby couples the optical with the acoustical part.

Next, we recall continuity and differentiability of the forward operators. For that purpose, we call h∈X a feasible direction at μ∈D(T), if there exists some ϵ>0 with μ+ϵh∈D(T).

**Theorem** **2** (Continuity and Differentiability).

(1) The operators $T_{i}$, $F_{i}$ and $H_{i}$ are sequentially continuous and Lipschitz-continuous.
(2) *For every μ∈D(T), the one-sided directional derivatives $T_{i}^{\prime }\left(\mu \right)\left(h\right)$, $F_{i}^{\prime }\left(\mu \right)\left(h\right)$ of $T_{i}$, $F_{i}$ at μ in any feasible direction h exist, and are given by*
  (13) $$\begin{array}{cc} T_{i}^{\prime }\left(\mu \right)\left(h\right) & =T\left(0,-\left(h_{a}+h_{s}-h_{s}K\right)T\left(\mu \right),\mu \right), \end{array}$$
  (14) $$\begin{array}{cc} F_{i}^{\prime }\left(\mu \right)\left(h\right) & =U_{\Lambda_{i},T_{i}}\left(h_{a}AT_{i}\left(\mu \right)+\mu_{a}A\left(T_{i}^{\prime }\left(\mu \right)\left(h\right)\right)\right)\;. \end{array}$$

**Proof.**  See [18]. ☐

Equations (13) and (14) define a bounded linear operator $F_{i}^{\prime }\left(\mu \right):X\to Y$, which we call the derivative of $F_{i}$ at μ∈D(T). Numerical minimization schemes actually require the adjoint of $F_{i}^{\prime }\left(\mu \right)$, which we compute next.

**Theorem** **3** (Adjoint of $F_{i}^{\prime }\left(\mu \right)$). Let i∈{1,…,N} and μ∈D(T). Furthermore, set $\Phi_{i}:=T_{i}\left(\mu \right)$ and let $\Phi_{i}^{*}$ denote the solution of the adjoint problem

(15) $$\begin{array}{c} \left(-\theta \;\cdot \;\nabla_{x}+\left(\mu_{a}+\mu_{s}-\sigma K\right)\right)\Phi_{i}^{*}=-A^{*}\left(\mu_{a}\left(U_{i}^{*}v\right)\right) \end{array}$$

with $\Phi_{i}^{*}{|}_{\Gamma_{+}}=0$. Then, $F_{i}^{\prime }{\left(\mu \right)}^{*}:Y\to X$ is given by

(16) $$F_{i}^{\prime }{\left(\mu \right)}^{*}v=\left[\begin{array}{c} A\left(\Phi_{i}^{*}\Phi_{i}\right)+\left(A\Phi_{i}\right)\left(U_{i}^{*}v\right)\\ A\left(I-K\right)\left(\Phi_{i}^{*}\Phi_{i}\right) \end{array}\right]\;.$$

**Proof.**  See [18]. ☐

Given data $v_{1},\ldots ,v_{N}\in Y$, most numerical schemes for QPAT use gradients of the partial data-fidelity terms $F_{I}:D\left(T\right)\to R$ for I⊆{1,…,N}, where

(17) $$F_{I}\left(\mu \right)=\sum_{i\in I}F_{i}\left(\mu \right)\;\;\mathrm{with}\;\;F_{i}\left(\mu \right):=\frac{1}{2}{∥F_{i}\left(\mu \right)-v_{i}∥}_{Y}^{2}\;.$$

By the chain rule, the gradient of $F_{I}$ is given by $\nabla F_{I}\left(\mu \right)=\sum_{i\in I}\nabla F_{i}\left(\mu \right)$ with $\nabla F_{i}\left(\mu \right)=F_{i}^{\prime }{\left(\mu \right)}^{*}\left(F_{i}\left(\mu \right)-v_{i}\right)$, where $F_{i}^{\prime }{\left(\mu \right)}^{*}$ can be computed by Theorem 3. Convergence of schemes such as the (stochastic) proximal gradient method considered in the following section require the Lipschitz continuity of $\nabla F_{I}$, which will be shown in the following theorem.

**Theorem** **4** (Lipschitz continuity of ∇*F*_*I*_) For any data $v_{1},\ldots ,v_{N}\in Y$ and any subset I⊆{1,…,N}, the map $\mu \mapsto \nabla F_{I}\left(\mu \right)$ is Lipschitz-continuous.

**Proof.**  Without loss of generality, we assume N=1, I={1} and write $v=v_{1}$, $F=F_{\left\{1\right\}}$, $T=T_{1}$, $U=U_{1}$, and v(μ)=F(μ)−v. For any μ∈D(T), let $T^{*}\left(\mu \right)$ denote the solution of (15) with v(μ) in place of *v*. Then, for any $\mu ,\tilde{\mu }\in D\left(T\right)$,

(18) $$\begin{array}{c} {∥\nabla F\left(\mu \right)-\nabla F\left(\tilde{\mu }\right)∥}_{X}^{2}\\ \;=∥A\left(T^{*}\left(\mu \right)T\left(\mu \right)\right)+\left(AT\left(\mu \right)\right)\left(U^{*}v\left(\mu \right)\right)-A\left(T^{*}\left(\tilde{\mu }\right)T\left(\tilde{\mu }\right)\right)-\left(AT\left(\tilde{\mu }\right)\right)\left(U^{*}v\left(\tilde{\mu }\right)\right){∥}_{L^{2}\left(\Omega \right)}^{2}\\ \;+{∥A\left(I-K\right)\left(T^{*}\left(\mu \right)T\left(\mu \right)\right)-A\left(I-K\right)\left(T^{*}\left(\tilde{\mu }\right)T\left(\tilde{\mu }\right)\right)∥}_{L^{2}\left(\Omega \right)}^{2}\;. \end{array}$$

For the second term in (18), we obtain

$$\begin{array}{cc} ∥A\left(I-K\right)(T^{*}\left(\mu \right) & T\left(\mu \right))-A\left(I-K\right)\left(T^{*}\left(\mu \right)T\left(\tilde{\mu }\right)\right)\\ + & A\left(I-K\right)\left(T^{*}\left(\mu \right)T\left(\tilde{\mu }\right)\right)-A\left(I-K\right)\left(T^{*}\left(\tilde{\mu }\right)T\left(\tilde{\mu }\right)\right){∥}_{L^{2}\left(\Omega \right)}\\ = & ∥A\left(I-K\right)\left(T^{*}\left(\mu \right)\left[T\left(\mu \right)-T\left(\tilde{\mu }\right)\right]\right)+A\left(I-K\right)\left(T\left(\tilde{\mu }\right)\left[T^{*}\left(\mu \right)-T^{*}\left(\tilde{\mu }\right)\right]\right){∥}_{L^{2}\left(\Omega \right)}\\ \leq & c_{1}∥A(I-K)∥{∥T^{*}\left(\mu \right)∥}_{W}L_{T}{∥\mu -\tilde{\mu }∥}_{X}+c_{1}∥A(I-K)∥{∥T(\tilde{\mu })∥}_{W}L_{T^{*}}{∥\mu -\tilde{\mu }∥}_{X}\\ \leq & 2c_{1}∥A(I-K)∥\max\left({∥T^{*}\left(\mu \right)∥}_{W},{∥T(\tilde{\mu })∥}_{W}\right)\max\left(L_{T^{*}},L_{T}\right){∥\mu -\tilde{\mu }∥}_{X}\;, \end{array}$$

where $L_{T^{*}}$ and $L_{T}$ denote the Lipschitz constants of $T^{*}$ and T, and $c_{1}$ is a constant. The difference $A\left(T^{*}\left(\mu \right)T\left(\mu \right)\right)\;-\;A\left(T^{*}\left(\tilde{\mu }\right)T\left(\tilde{\mu }\right)\right)$ in the first term in (18) is estimated in a similar manner. Furthermore, we have

$$\begin{array}{c} ∥\left(AT\left(\mu \right)\right)\left(U^{*}v\left(\mu \right)\right)-\left(AT\left(\tilde{\mu }\right)\right)\left(U^{*}v\left(\mu \right)\right)+\left(AT\left(\tilde{\mu }\right)\right)\left(U^{*}v\left(\mu \right)\right)-\left(AT\left(\tilde{\mu }\right)\right)\left(U^{*}v\left(\tilde{\mu }\right)\right){∥}_{L^{2}\left(\Omega \right)}\\ \leq ∥\left(U^{*}v\left(\mu \right)\right)A\left[T\left(\mu \right)-T\left(\tilde{\mu }\right)\right]{∥}_{L^{2}\left(\Omega \right)}+{∥AT\left(\tilde{\mu }\right)\left(U^{*}\left(v\left(\mu \right)-v\left(\tilde{\mu }\right)\right)\right)∥}_{L^{2}\left(\Omega \right)}\;. \end{array}$$

Noting that A, U and $U^{*}$ are linear and bounded, Theorem 2 and the computations above yield the Lipschitz continuity of ∇F. ☐

## 3. The Stochastic Proximal Gradient Method for QPAT

### 3.1. Formulation of the Inverse Problem

The inverse problem of multi-source QPAT consists in finding $\mu^{★}\in X$ from measured data

(19) $$\begin{array}{c} v_{i}=F_{i}\left(\mu^{★}\right)+z_{i}\;\;\mathrm{for}\;i=1,\ldots ,N\;. \end{array}$$

Here, $\mu^{★}=\left(\mu_{a}^{★},\mu_{s}^{★}\right)$ are the unknowns to be estimated, $z_{i}$ are the unknown error vectors, and $v_{1},\ldots ,v_{N}$ are the given noisy data. Using the notation

$$\begin{array}{cc} v & :=\left(v_{1},\ldots ,v_{N}\right)\in Y^{N},\\ F & :=\left(F_{1},\ldots ,F_{N}\right):D\left(T\right)\to Y^{N}, \end{array}$$

we can write (19) in the alternative form

(20) $$\begin{array}{c} \mathrm{Estimate}\;\mu^{*}\in X\;\mathrm{from}\;v=F(\mu^{*})+z\;. \end{array}$$

Here, $z\in Y^{N}$ denotes the error vector.

There are, at least, two different strategies to address such an inverse problem: Tikhonov type regularization on the one and iterative methods on the other hand. In this section, we give an overview of such methods. In particular, we describe proximal stochastic gradient methods (for minimizing the Tikhonov functional), which seem particularly well suited for multi-source QPAT but have not been investigated yet for that purpose.

### 3.2. Tikhonov Regularization in QPAT

In this section, we consider a quadratic Tikhonov regularization term for solving (19). Let

$$\begin{array}{cc} L:D(L)\subseteq X\to Z & :\mu \mapsto L\mu \; \end{array}$$

be a linear, densely defined, and possibly unbounded operator between X and another Hilbert space $\left(Z,{\langle \;\;\cdot \;\;,\;\;\cdot \;\;\rangle }_{Z}\right)$ and set D:=D∩D(L). In this context, any element $\mu^{+}\in D$ with $∥L\mu^{+}∥=\min\left\{∥L\mu ∥|F\left(\mu \right)=v\right\}$ is called an $∥L(\;\;\cdot \;\;)∥$-minimizing solution of Fμ=v. Tikhonov regularization with regularization term $\frac{1}{2}{∥L\mu ∥}_{Z}^{2}$ consists in computing a minimizer of the generalized Tikhonov functional $T_{v,\lambda }:X\to R\cup \left\{\infty \right\}$, defined by

(21) $$T_{v,\lambda }\left(\mu \right):=\left\{\begin{array}{cc} \frac{1}{2}{∥F(\mu )-v∥}^{2}+\frac{\lambda }{2}{∥L\mu ∥}^{2}, & \mathrm{if}\;\mu \in D,\\ \infty , & \mathrm{otherwise}\;. \end{array}\right.$$

Here, λ>0 denotes the regularization parameter that acts as a trade-off between the data fitting term and stability.

**Theorem** **5** (Well-posedness and convergence).

(1) For any v∈Y and any λ>0, the Tikhonov functional $T_{\lambda ,v}$ has at least one minimizer.
(2) *Let v∈ran(F), ${\left(\delta_{m}\right)}_{m\in N}\in {\left(0,\infty \right)}^{N}$, ${\left(v^{m}\right)}_{m\in N}\in Y^{N}$ with $∥v-v^{m}∥\leq \delta_{m}$. Suppose further that ${\left(\lambda_{m}\right)}_{m\in N}\in {\left(0,\infty \right)}^{N}$ satisfies $\lambda_{m}\to 0$ and $\delta_{m}^{2}/\lambda_{m}\to 0$ as m→∞. Then:*
  ■ Every sequence ${\left(\mu^{m}\right)}_{m\in N}$ with $\mu^{m}\in \arg\;\min T_{v^{m},\lambda_{m}}$ has a weakly converging subsequence.
  ■ The limit of every weakly convergent subsequence of ${\left(\mu^{m}\right)}_{m\in N}$ is an $∥L(\;\;\cdot \;\;)∥$-minimizing solution of Fμ=v.
  ■ If the $∥L(\;\;\cdot \;\;)∥$-minimizing solution of Fμ=v is unique and denoted by $\mu^{+}$, then $\left(\mu^{m}\right)⇀\mu^{+}$.

**Proof.**  See [18]. ☐

### 3.3. The Proximal Stochastic Gradient Algorithm for QPAT

Depending on the particular choice of L, the Tikhonov functional (21) may be ill-conditioned. To address this issue in [18], we proposed the proximal gradient algorithm for minimizing (21), which is a very flexible algorithm for minimizing functionals of the form F+G, where *F* is smooth and *G* is convex (see, for example, [35,36]). Here, we extend the approach to the proximal stochastic gradient algorithm. Additionally, we propose computing the proximal step using Dykstra’s projection algorithm.

■ **Proximal gradient algorithm:** The proximal gradient algorithm is a splitting method that iteratively computes explicit gradient steps for *F* and implicit proximal steps for *G*. In our context, we take *F* as the data fidelity term and

(22) $$G\left(\mu \right)=G_{\lambda }\left(\mu \right):=g_{\lambda }\left(\mu \right)+\chi_{D}\left(\mu \right):=\frac{\lambda }{2}{∥L\mu ∥}^{2}+\chi_{D}\left(\mu \right)\;,$$

where $\chi_{D}$ is the characteristic function taking the value zero inside D and ∞ outside. The proximal gradient algorithm for minimizing the QPAT-Tikhonov functional (21) reads

(23) $$\mu^{k+1}={\mathrm{prox}}_{s_{k}G_{\lambda }}\left(\mu^{k}-s_{k}\sum_{i=1}^{N}\nabla F_{i}\left(\mu^{k}\right)\right)\;.$$

Here, ${\mathrm{prox}}_{s_{k}G_{\lambda }}:X\to D$ denotes the proximal mapping corresponding to the functional $s_{k}G_{\lambda }$,

(24) $${\mathrm{prox}}_{s_{k}G_{\lambda }}\left(x\right)=\arg\;\min\left\{\frac{1}{2}{∥x-(\;\;\cdot \;\;)∥}^{2}+s_{k}G_{\lambda }\right\}\;.$$

Furthermore, $\nabla F_{i}\left(\mu^{k}\right)$ is the gradient of the *i*-th data fidelity term computed in Theorem 3.

■ **Dykstra’s projection algorithm:** The constraint quadratic optimization problem (24) can efficiently be solved by a proximal variant of Dykstra’s projection algorithm [35,36,53]. For that purpose, we write $s_{k}G_{\lambda }=\chi_{D}+g$ with $g\left(x\right):=\frac{s_{k}\lambda }{2}{∥Lx∥}^{2}$. Setting $x_{0}=\mu$, $p_{0}=0$ and $q_{0}=0$, Dykstra’s projection algorithm for (24) reads, for m∈N,

(25) $$\begin{array}{cc} y_{m} & ={\mathrm{prox}}_{g}\left(x_{m}+p_{m}\right), \end{array}$$

(26) $$\begin{array}{cc} x_{m+1} & =P_{D}\left(y_{m}+q_{m}\right), \end{array}$$

(27) $$\begin{array}{cc} p_{m+1} & =x_{m}+p_{m}-y_{m}, \end{array}$$

(28) $$\begin{array}{cc} q_{m+1} & =y_{m}+q_{m}-x_{m+1}. \end{array}$$

Both proximal mapping in (25) and the projection in (26) can be computed explicitly. In fact, one readily verifies that

(29) $$\begin{array}{cc} {\mathrm{prox}}_{g}\left(x\right) & ={\left(I_{X}+s_{k}\lambda \;L^{*}L\right)}^{-1}x, \end{array}$$

(30) $$\begin{array}{cc} P_{D}\left(\mu \right) & =\min\left\{\bar{\mu },\max\left\{0,\mu \right\}\right\}\;. \end{array}$$

Here, $I_{X}$ is the identity operator on X and $P_{D}$ the projection onto D.

■ **Proximal stochastic gradient algorithm:** The methods described so far require in any iterative step the computation of the full gradient

$$\nabla F\left(\mu \right)=\sum_{i=1}^{N}\nabla F_{i}\left(\mu \right)\;\;\mathrm{with}\;\;\nabla F_{i}\left(\mu \right)=F_{i}^{\prime }{\left(\mu \right)}^{*}\left(F_{i}\left(\mu \right)-v_{i}\right)\;.$$

The evaluation of each $\nabla F_{i}\left(\mu \right)$ requires the solution of the RTE and an adjoint problem and therefore is quite time-consuming. For multi-source QPAT, where N>1, a significant acceleration may be obtained by a Kaczmarz strategy, where in each iterative step only one of the summands $\nabla F_{i}\left(\mu \right)$ is used. The resulting proximal stochastic gradient method for minimizing the Tikhonov functional (21) in QPAT reads

(31) $$\mu^{k+1}={\mathrm{prox}}_{s_{k}G_{\lambda }}\left(\mu^{k}-s_{k}\nabla F_{i(k)}\left(\mu^{k}\right)\right)\;,$$

where $i\left(k\right)\in \left\{1,\ldots ,N\right\}$ is selected randomly for the update in the *k*-th iteration. Furthermore, ${\mathrm{prox}}_{s_{k}G_{\lambda }}$ is the proximal mapping of $s_{k}G_{\lambda }$ that can be computed by Dykstra algorithm (25)–(28) and $\nabla F_{i}\left(\mu^{k}\right)$ is the gradient of the *i*-th data fidelity term that can be computed by Theorem 3.

One can also incorporate a block-iterative (or mini-batch) strategy in the stochastic gradient method, meaning that a small subset of {1,…,N} of equations is used per iteration instead of a single one. Such a variant could be especially useful in the case of a large number of different illumination patterns. For more details about stochastic gradient methods, see [37,38,39,40,41,42] and the references therein. Note that, in general, convergence of stochastic gradient methods requires asymptotically vanishing step size [42].

### 3.4. Iterative Regularization Methods

An alternative class of algorithms to address nonlinear inverse problems are iterative techniques. The most basic iterative method for solving the nonlinear inverse problem v=F(μ) is the Landweber iteration. In the case that the domain of definition D is a proper subset, we have to combine the Landweber iteration with a projection step onto D as presented in this subsection. The projected Landweber iteration applied to multi-source QPAT reads

(32) $$\mu^{k+1}=P_{D}\left(\mu^{k}-s_{k}\sum_{i=1}^{N}\nabla F_{i}\left(\mu^{k}\right)\right)\;,$$

where $\nabla F_{i}$ is the gradient of $F_{i}$ (see Equation (17)), and $P_{D}\left(\mu \right)=\min\left\{\bar{\mu },\max\left\{0,\mu \right\}\right\}$ denotes the projection onto D. In Tikhonov regularization, the regularity of solutions is enforced by an explicitly included penalty. In opposition to that, in iterative regularization methods, a stabilization effect is enforced by early stopping of the iteration. A common stopping rule is the discrepancy principle, where iteration is stopped at the smallest index k∈N satisfying $∥v-F(\mu^{k})∥\leq \tau \delta ,$ where δ is an estimate for the noise and τ≥1. Formally, the projected Landweber iteration (32) arises as a special case of the proximal gradient iteration (23) for minimizing the Tikhonov functional, where the regularization parameter is taken as λ=0 and where the proximal mapping (24) reduces to the orthogonal projection onto D.

In a similar manner, one can also use a stochastic version of the projected Landweber iteration. Using the loping strategy of [43,44,45] in order to stabilize the iterative process, the resulting projected loping Landweber–Kaczmarz iteration reads

(33) $$\begin{array}{cc} \mu^{k+1} & =P_{D}\left(\mu^{k}-s_{k}\omega_{k}\nabla F_{i(k)}\left(\mu^{k}\right)\right)\;, \end{array}$$

(34) $$\begin{array}{cc} \omega_{k} & :=\left\{\begin{array}{cc} 1, & ∥F_{i(k)}\left(\mu^{k}\right)-v_{i(k)}{∥}_{X}>\tau \delta_{i(k)},\\ 0, & \mathrm{otherwise}. \end{array}\right.\; \end{array}$$

Here, $i\left(k\right)\in \left\{1,\ldots ,N\right\}$ for any k∈N may be randomly selected, τ>1 is an appropriately chosen positive constant, $\nabla F_{i}\left(\;\;\cdot \;\;\right)$ is the gradient of the *i*-th data fidelity term computed in Theorem 3 and $P_{D}\left(\mu \right)=\min\left\{\bar{\mu },\max\left\{0,\mu \right\}\right\}$ denotes the projection onto D. The iteration (33), (34) terminates if $∥F_{i}\left(\mu^{k}\right)-v_{i}∥\leq \tau \delta$ for all $i\in \left\{1,\ldots ,N\right\}$. It is worth mentioning that, for noise free data, we have $\omega_{k}=1$ for all *k* and, therefore, in this special situation, the iteration becomes $\mu^{k+1}=P_{D}\left(\mu^{k}-s_{k}\nabla F_{i(k)}\left(\mu^{k}\right)\right)$, which formally arises from the proximal stochastic gradient method (31) with λ=0. A convergence analysis of the loping Landweber–Kaczmarz method can be found in [43,44].

## 4. QPAT as Multilinear Inverse Problem

Since the RTE is time-consuming to solve, we are looking for a suitable reformulation of the inverse problem in multi-source QPAT avoiding computation of a solution of the RTE in each iterative step. In this paper, we propose to write (19) as a multilinear inverse problem, where we add the RTE as a constraint instead of explicitly including its solution. The new formulation will again be addressed by Tikhonov regularization and proximal stochastic gradient methods.

### 4.1. Reformulation as Multilinear Inverse Problem

Recall the forward problem of QPAT governed by the RTE (6). With the abbreviation $M\left(\mu \right):=\theta \;\cdot \;\nabla_{x}+\mu_{a}+\mu_{s}\left(I-K\right)$, the RTE can be written in compact form M(μ)Φ=q, where $\mu =(\mu_{a},\mu_{s})$ is the unknown parameter pair. In the case of exact data, the multi-source problem in QPAT (19) then can be reformulated as the problem of finding the tuple $z:=\left(\mu ,{\left(\Phi_{i},H_{i}\right)}_{i=1}^{N}\right)\in D\times {\left(W\times L^{2}\left(\Omega \right)\right)}^{N}$ such that

(35) $$\begin{array}{ccc} M\left(\mu \right)\Phi_{i} & =q_{i} & \;\mathrm{for}\;i=1,\ldots ,N,\\ H_{i} & =\mu_{a}\;A\Phi_{i} & \;\mathrm{for}\;i=1,\ldots ,N,\\ v_{i} & =U(H_{i}) & \;\mathrm{for}\;i=1,\ldots ,N\;. \end{array}$$

Here, the index *i* indicates the *i*-th illumination, and $q_{i}\in Q$, $\Phi_{i}\in W$, $H_{i}\in L^{2}\left(\Omega \right)$, $v_{i}\in Y$ are the corresponding source, photon density, heating and acoustical data, respectively, and $A\Phi_{i}=\int_{S^{d-1}}\Phi_{i}\left(\;\;\cdot \;\;,\theta \right)d\theta$ is the averaging operator. We call (35) and resulting formulations below the multilinear (MULL) formulation of QPAT.

### 4.2. Application of Tikhonov Regularization

In the case that the data $v_{i}$ are only known approximately, we use Tikhonov regularization for the stable solution of (35). For that purpose, we approximate (35) by the constrained optimization problem

(36) $$\begin{array}{c} \min_{{\left(\mu ,\Phi_{i},H_{i}\right)}_{i=1}^{N}}\left(\frac{1}{2}\sum_{i=1}^{N}{∥v_{i}-U\left(H_{i}\right)∥}^{2}+\frac{\lambda }{2}{∥L(\mu )∥}^{2}+\chi_{D}\left(\mu \right)\right),\\ s.t.\;\left\{\begin{array}{c} M\left(\mu \right)\Phi_{i}=q_{i},\\ H_{i}=\mu_{a}\;A\Phi_{i}\;\;\mathrm{for}\;i=1,\ldots ,N\;. \end{array}\right. \end{array}$$

Here, the operator $L\mu =(L_{a}\mu_{a},L_{s}\mu_{s})$ is possibly unbounded, $\frac{\lambda }{2}{∥L\left(\mu \right)∥}^{2}$ is the regularization term and λ>0 the regularization parameter. Note that (36) is equivalent to (21) and therefore the well-posedness and convergence results of Theorem 5 apply to (36) as well.

The constrained optimization problem (36) proposed in this paper can be addressed by various solution methods, for example using penalty methods or augmented Lagrangian techniques [54]. In this paper, we use a penalty approach for solving (36) where the constraints are included as penalty term. To simplify notation, we introduce the unconstraint functionals

(37) $$\begin{array}{cc} J^{\left(i\right)}\left(z\right):=\frac{a_{1}}{2}{∥M\left(\mu \right)\Phi_{i}-q_{i}∥}^{2}+\frac{a_{2}}{2}{∥\mu_{a}\;A\Phi_{i}-H_{i}∥}^{2} & \\ & +\frac{a_{3}}{2}{∥v_{i}-U\left(H_{i}\right)∥}^{2}+\frac{\lambda }{2}{∥L(\mu )∥}^{2}+\chi_{D}\left(\mu \right)\;, \end{array}$$

for certain parameters $a_{1},a_{2},a_{3}>0$ and $z_{i}:=\left(\mu ,\Phi_{i},H_{i}\right)\in Q\times W\times L^{2}\left(\Omega \right)$. The sum of the unconstraint functionals (37) over all illuminations will actually be minimized in our numerical implementations. For that purpose, we define

$$\begin{array}{cc} J_{1}^{\left(i\right)}\left(z\right) & =\frac{1}{2}{∥M\left(\mu \right)\Phi_{i}-q_{i}∥}^{2},\\ J_{2}^{\left(i\right)}\left(z\right) & =\frac{1}{2}{∥\mu_{a}\;A\Phi_{i}-H_{i}∥}^{2},\\ J_{3}^{\left(i\right)}\left(z\right) & =\frac{1}{2}{∥v_{i}-U\left(H_{i}\right)∥}^{2},\\ J_{4}^{\left(i\right)}\left(z\right) & =\frac{1}{2}{∥L(\mu )∥}^{2}\;. \end{array}$$

Then, we have $J^{\left(i\right)}\left(z\right)=\sum_{\ell =1}^{4}a_{\ell }J_{\ell }^{\left(i\right)}\left(z\right)+\chi_{D}\left(\mu \right)$. For the approximate solution of (36), we minimize the unconstrained functional $J\left(z\right)=\sum_{i=1}^{4}J^{\left(i\right)}\left(z\right)$, which can be written in the forms

(38) $$\begin{array}{cc} J(z) & =\sum_{i=1}^{N}\sum_{\ell =1}^{4}a_{\ell }J_{\ell }^{\left(i\right)}\left(z\right)+\chi_{D}\left(\mu \right), \end{array}$$

(39) $$\begin{array}{cc} J(z) & =\sum_{i=1}^{N}\sum_{\ell =1}^{3}a_{\ell }J_{\ell }^{\left(i\right)}\left(z\right)+\frac{\lambda }{2}{∥L(\mu )∥}^{2}+\chi_{D}\left(\mu \right). \end{array}$$

(Here and below, we also write $a_{4}=\lambda$, if it simplifies notation.) The formulations (38) as well as (39) can be solved by various optimization techniques. In particular, as the functionals are given as the sum of simpler terms, the stochastic (proximal) gradient method is particularly appealing.

### 4.3. Solution of the MULL Formulation of QPAT Using Stochastic Gradient Methods

For solving QPAT in the novel MULL formulation (35), we use stochastic gradient methods similar to previous sections. For that purpose, we require the gradients (determining the steepest descent directions) of the individual functionals $J_{\ell }^{\left(i\right)}\left(z_{i}\right)$ with respect to $z_{i}=\left(\mu ,\Phi_{i},H_{i}\right)$, which are given as

(40) $$\begin{array}{cc} \nabla_{\mu_{a}}J_{1}^{\left(i\right)}\left(z\right) & =\Phi_{i}\left(M\left(\mu \right)\Phi_{i}-q_{i}\right), \end{array}$$

(41) $$\begin{array}{cc} \nabla_{\mu_{s}}J_{1}^{\left(i\right)}\left(z\right) & =\left(I-K\right)\Phi_{i}\left(M\left(\mu \right)\Phi_{i}-q_{i}\right), \end{array}$$

(42) $$\begin{array}{cc} \nabla_{\Phi_{i}}J_{1}^{\left(i\right)}\left(z\right) & =M\left(\mu \right)(M\left(\mu \right)\Phi_{i}-q_{i}), \end{array}$$

(43) $$\begin{array}{cc} \nabla_{\mu_{a}}J_{2}^{\left(i\right)}\left(z\right) & =\left(A\Phi_{i}\right)\left(\mu_{a}A\Phi_{i}-H_{i}\right), \end{array}$$

(44) $$\begin{array}{cc} \nabla_{H_{i}}J_{2}^{\left(i\right)}\left(z\right) & =-(\mu_{a}A\Phi_{i}-H_{i}), \end{array}$$

(45) $$\begin{array}{cc} \nabla_{\Phi_{i}}J_{2}^{\left(i\right)}\left(z\right) & =A^{*}\left[\mu_{a}\left(\mu_{a}A\Phi_{i}-H_{i}\right)\right], \end{array}$$

(46) $$\begin{array}{cc} \nabla_{H_{i}}J_{3}^{\left(i\right)}\left(z\right) & =-U^{T}\left(v_{i}-U\left(H_{i}\right)\right), \end{array}$$

(47) $$\begin{array}{cc} \nabla_{\mu_{a}}J_{4}^{\left(i\right)}\left(z\right) & =L_{a}^{*}L_{a}\mu_{a}, \end{array}$$

(48) $$\begin{array}{cc} \nabla_{\mu_{s}}J_{4}^{\left(i\right)}\left(z\right) & =L_{s}^{*}L_{s}\mu_{s}.\; \end{array}$$

(All other partial gradients are vanishing.) In the following, let *N* be the number of illuminations, write $z=(\mu_{a},\mu_{s},{\left(\Phi_{i},H_{i}\right)}_{i=1}^{N})$ and let ${\left(s_{k}\right)}_{k\in N}$ be a sequence of step sizes. In this paper, we propose the following instances of the stochastic proximal gradient method for QPAT based on the multilinear formulation (35).

■ **MULL-projected stochastic gradient algorithm:** Here, we consider the form (38). For any iteration index k∈N choose i(k)∈{1,…,N} and ℓ(k)∈{1,…,4} and define the sequence of iterates ${\left(z^{k}\right)}_{k\in N}$ by

(49) $$z^{k+1}=\left(P_{D}\times I\right)\left(z^{k}-s_{k}\nabla J_{\ell (k)}^{\left(i(k)\right)}\left(z^{k}\right)\right).$$

Here, the mapping $P_{D}\times I$ is the proximal mapping corresponding to $z\mapsto \chi_{D}\left(\mu \right),$ which equals the projection $P_{D}$ in the μ component and equals the identity I in the other components.

■ **MULL-proximal stochastic gradient algorithm:** Here, we consider the form (39). For any iteration index k∈N choose i(k)∈{1,…,N} and ℓ(k)∈{1,…,3} and define sequence of iterates ${\left(z^{k}\right)}_{k\in N}$ by

(50) $$z^{k+1}={\mathrm{prox}}_{s_{k}G_{\lambda }}\left(z^{k}-s_{k}\nabla J_{\ell (k)}^{\left(i(k)\right)}\left(z^{k}\right)\right)\;.$$

The second step implements the proximal mapping of $z\mapsto s_{k}G_{\lambda }\left(\mu \right)$ with $G_{\lambda }\left(\mu \right)=\frac{\lambda }{2}{∥L(\mu )∥}^{2}+\chi_{D}\left(\mu \right)$. As in the previous section, this can be computed with Dykstra’s projection algorithm (25)–(28).

For better scaling, in our actual numerical implementation, we replace the scalar step sizes $s_{k}$ by the adaptive step size rule

(51) $$s_{k}^{i,\ell }:=\arg\;\min\left\{z^{k}-t\nabla J_{\ell }^{\left(i\right)}\left(z^{k}\right)|t\in R\right\}\;.$$

Note that computing such step sizes does barely increase the computational time of the stochastic gradient method, since all involved calculations are anyhow necessary for computing the gradient for the iterative update. In opposition to that, calculating a similar adaptive step size for the algorithms proposed in Section 3 would require evaluation of the forward operators $F_{i}$ and therefore would significantly increase the computation time. This might be seen as an additional advantage of the novel MULL formulation (35) and its regularized version (36).

## 5. Numerical Simulations

For the Tikhonov approach to multi-source QPAT, the radiative transfer equation is numerically solved by a streamline diffusion finite element method. Solving the RTE is required to evaluate the forward operator F and the gradient ∇F of the data fidelity term in every iterative step. For the alternative multilinear approach, these calculations are not necessary. However, the application of the transport operator to Φ has to be calculated for every update of $J_{1}$. The simulations are performed on the square domain $\Omega ={\left[-1\mathrm{cm},1\mathrm{cm}\right]}^{2}$, where the absorption and the scattering coefficient are supported.

### 5.1. Numerical Solution of the RTE

Employing a finite element scheme, we derive the weak formulation of Equation (6) by integrating against a test function $w:\Omega \times S^{1}\to R$ and replacing the exact solution Φ by a linear combination in the finite element space $\Phi^{\left(h\right)}=\sum_{i=1}^{N_{h}}c_{i}^{\left(h\right)}\psi_{i}^{\left(h\right)}\left(x,\theta \right)$ as in [18]. Here, the basis function $\psi_{i}^{\left(h\right)}\left(x,\theta \right)$ is the product of a basis function in space and a basis function in velocity. The spatial domain is triangulated uniformly with mesh size *h* and $P_{1}$-Lagrangian element function for the spatial and velocity domain. By choosing the test function $w\left(x,\theta \right)=\sum_{j=1}^{N_{h}}w_{j}\left(\psi_{j}\left(x,\theta \right)+D\left(x,\theta \right)\theta \;\cdot \;\nabla_{x}\psi_{j}\left(x,\theta \right)\right)$ with streamline diffusion coefficient D(x,θ), we obtain

(52) $$\begin{array}{cc} \int_{\Omega }\int_{S^{1}}\left(D\theta \;\cdot \;\psi_{i}-\psi_{i}\right)\theta \;\cdot & \nabla_{x}\psi_{j}d\theta dx+\int_{\Gamma_{+}}\left|\theta \;\cdot \;\nu \right|d\sigma \\ & +\int_{\Omega }\int_{S^{1}}\left(\mu_{a}+\mu_{s}-\mu_{s}K\right)\left(\psi_{j}+D\theta \;\cdot \;\nabla_{x}\psi_{j}\right)\psi_{i}d\theta dx=\int_{\Gamma_{-}}\left|\theta \;\cdot \;\nu \right|\psi_{i}\psi_{j}d\sigma \;. \end{array}$$

Equation (52) yields a system of linear equations $M^{\left(h\right)}c^{\left(h\right)}=b^{\left(h\right)}$, where evaluating the left-hand side of (52) provides the entries of $M^{\left(h\right)}$, the right-hand side gives the components of vector $b^{\left(h\right)}$. Note that the sparsity of matrix $M^{\left(h\right)}$ is low and solving the linear system for the Tikhonov approach is very time-consuming. On the other hand, the solution via the MULL formulation requires only a matrix vector multiplication, since in this case $\Phi^{\left(h\right)}$ is an independent variable. Thus, only the application to Φ has to be calculated and the transport equation does not need to be solved.

### 5.2. Test Scenario for Multiple Illumination

**Figure 2:**
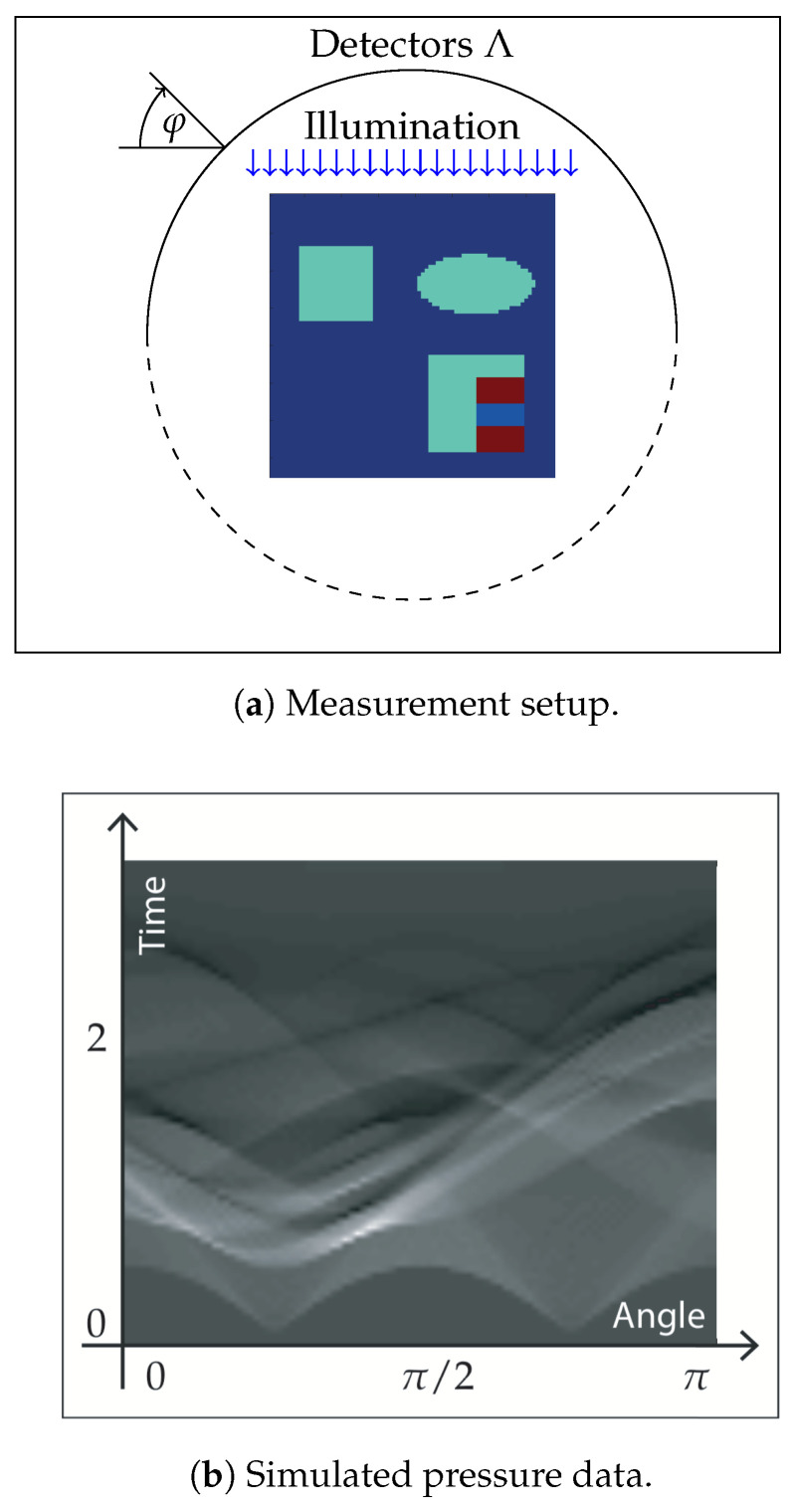
(**a**) The phantom is defined on the square $\Omega ={\left[-1\mathrm{cm},1\mathrm{cm}\right]}^{2}$ and the acoustic pressure is measured on a semi-circle on the side of the illumination; (**b**) the simulated pressure correspond to the phantom and the illumination in (a) and are represented as gray scale density.

The sample is illuminated in orthogonal direction at the boundaries of $\Omega ={\left[-1\mathrm{cm},1\mathrm{cm}\right]}^{2}$. In our simulations, we use N=4 homogenous illuminations and no internal sources. The illuminations are applied separately from each side (left, right, top and bottom) with acoustic data measured on a half circle on the same side as the illumination (see Figure 2). For the scattering kernel, we use the two-dimensional Henyey–Greenstein kernel,

$$k\left(\theta ,\theta^{\prime }\right):=\frac{1}{2\pi }\frac{1-g^{2}}{1+g^{2}-2g\cos\left(\theta \;\cdot \;\theta^{\prime }\right)}\;\;\mathrm{for}\;\theta ,\theta^{\prime }\in S^{1}\;,$$

where the anisotropy factor is chosen as g=0.5 in all our experiments.

For the simulated data, we choose a spatial mesh size 2/100, in order to discretize the velocity direction the unit circle is divided in 64 subintervals. In order to avoid inverse crime, for the reconstruction, we use a different spatial mesh size h=2/80 and use $N_{\theta }=48$ velocity directions. Calculating the simulated data corresponds to evaluating the forward operators $F_{i}$ with perpendicular boundary illumination constant along one side of the boundary square, $q_{o}\left(x,\theta \right)=\delta \left(\theta -\theta_{i}\right)\chi_{i}\left(x\right)1\;mJ\;{\mathrm{cm}}^{-1}$, where δ is an approximation of the Dirac delta function and $\chi_{i}$ the indicator function of side *i* of Ω. In this way, we simulate data

$$v_{i}=U\circ H_{i}\left(\mu \right)+z_{i}^{\mathrm{noise}}\;\;\mathrm{for}\;i=1,\ldots ,4\;.$$

**Figure 3:**
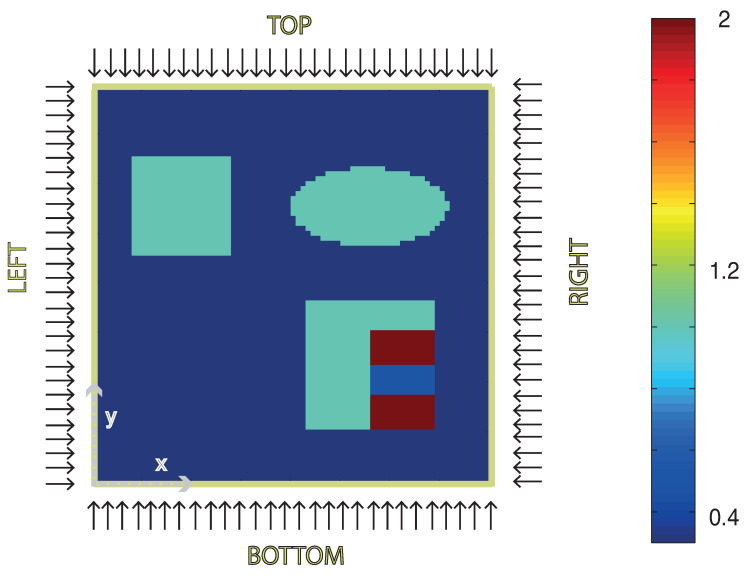
Absorption coefficient distribution of the tissue sample used for the numerical examples. Background absorption of the tissue is taken as $\mu_{a}=0.3\;{\mathrm{cm}}^{-1}$, the blue obstacles have $\mu_{a}=1\;{\mathrm{cm}}^{-1}$ and the red stripes $\mu_{a}=2\;{\mathrm{cm}}^{-1}$. The area between the red stripes has absorption coefficient $\mu_{a}=0.5\;{\mathrm{cm}}^{-1}$. The scattering coefficient is constant in the whole sample and chosen to be $\mu_{s}=3\;{\mathrm{cm}}^{-1}$. Illuminations are applied consecutively from top, right, bottom and left. The corresponding boundary sources are given by $q_{o}\left(x,\theta \right)=\delta \left(\theta -\theta_{i}\right)\chi_{i}\left(x\right)1\;mJ\;{\mathrm{cm}}^{-1}$. The *x*- and *y*-axis cover [−1cm,1cm].

Thereby, the heating operator is computed numerically by solving the RTE as described in Section 5.1. The wave operator U is evaluated by straightforward discretization of the well-known explicit formulas for (10) that can be found, for example, in [55,56]. In the following, we present results for exact data (where $z_{i}^{\mathrm{noise}}=0$) as well as for noisy data. For the noisy data case, we add 0.5% random noise to the simulated data, i.e., we take the maximum value of the simulated pressure and add white noise $z_{i}^{\mathrm{noise}}$ with a standard deviation of 0.5% of that maximal value. The phantom, the setup and the simulated data for one of the four illuminations (top) are shown in Figure 2 and Figure 3.

### 5.3. Numerical Results

For regularizing the absorption and scattering coefficient, we make use of Laplace regularization and choose $L_{a}=\Delta$ and $L_{s}=100\Delta$, respectively. We assume that the coefficient μ is known at the boundary of Ω and is therefore used as the starting value of our iterative schemes. Furthermore, we use the boundary value of μ for regularization; that is, we implement it in the Dykstra projection procedure (26) by iteratively projecting on the known boundary value. In the following, we discuss the methods that we have outlined in the previous section.

■ **Standard formulation of QPAT (19):** We assume that the scattering coefficient is known and we restrict ourself to reconstructing the absorption coefficient. Then, the proximal gradient and proximal stochastic gradient algorithm, respectively, read

(53) $$\begin{array}{cc} \mu_{a}^{k+1} & ={\mathrm{prox}}_{s_{k}G_{\lambda }}\left(\mu_{a}^{k}-\frac{s_{k}}{4}\sum_{i=1}^{4}\nabla F_{i}\left(\mu_{a}^{k},\mu_{s}\right)\right), \end{array}$$

(54) $$\begin{array}{cc} \mu_{a}^{k+1} & ={\mathrm{prox}}_{s_{k}G_{\lambda }}\left(\mu_{a}^{k}-s_{k}\nabla F_{i(k)}\left(\mu_{a}^{k},\mu_{s}\right)\right)\;. \end{array}$$

In contrast, to the full proximal gradient algorithm, the proximal stochastic gradient algorithm avoids evaluating the full gradient ∇F, but selects randomly an illumination number i∈{1,…,4} for each iterative step. Because of formula (16), each iteration of the above procedures requires the calculation of the solution of the radiative transfer equation Φ as well of its adjoint $\Phi^{*}$.

**Figure 4:**
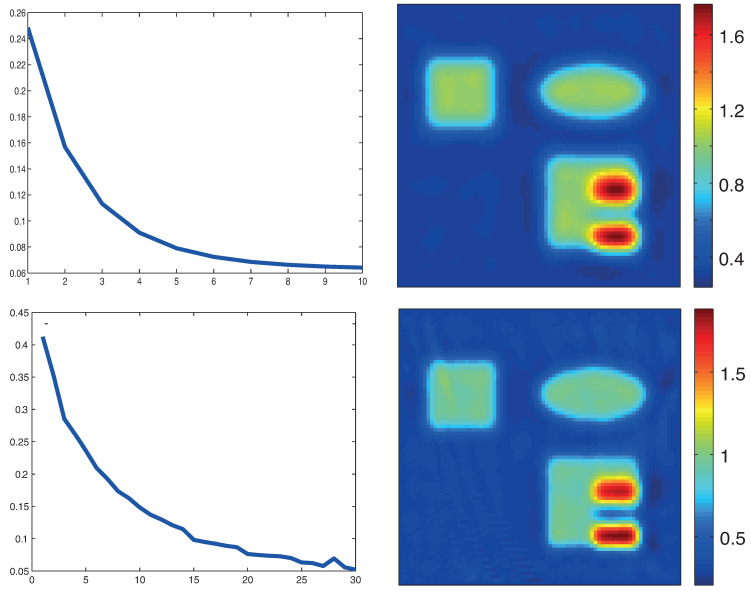
Reconstruction results based on standard formulation (19). **Top**: proximal gradient method; **Bottom**: proximal stochastic gradient method. The left images show the relative reconstruction errors of the reconstructed absorption coefficient as a function of the number of iterations, whereas the right pictures show the result after the final iteration. (The phantom is as described in Figure 3.)

The top row in Figure 4 shows reconstruction result for the absorption coefficient using the original formulation with the proximal gradient method with $\lambda =2\times 10^{-8}$ and 10 iterative steps. The left picture shows the relative error $∥\mu_{a}-\mu_{a}^{k}∥/∥\mu_{a}∥$. Note that, in this case, solutions of the RTE and its adjoint have to be computed for four illuminations per iterative step. The reconstruction results in the bottom row in Figure 4 are obtained by the proximal stochastic gradient method with $\lambda =2\times 10^{-7}$. The regularization parameters λ have been selected empirically as a trade-off between stability and accuracy. The total number of iterations is taken as 30. In each iteration, a illumination pattern is chosen randomly and the computation of RTE and its adjoint is executed only for this single illumination. Therefore, the computational effort for the proximal stochastic gradient method is approximately 3/4 of the proximal gradient algorithm using full gradients. For the algorithms based on the standard formulation (19), calculating adaptive step sizes similar to (51) is time-consuming as this requires another evaluation of the forward operator $F_{i}$ and therefore another solution of the RTE. Therefore, we simply use a constant step size rule; in our numerical experiments, it turned out that $s_{k}=0.5$ is a suitable choice.

■ **Novel MULL formulation of QPAT (35):** The multilinear approach overcomes the problem of solving the RTE by minimizing (38) or (39). In both cases, one selects an arbitrary functional and performs a steepest descent step, resulting in an iterative scheme for the variables $\mu_{a}$, $\mu_{s}$, Φ and *H*. Recall that none of the partial gradients (40)–(48) requires solving the RTE (which is the most time-consuming part for the standard formulation of QPAT). In each iterative step, we take a random illumination number i∈{1,…,4} and a random functional number ℓ∈{1,2,3}. The gradient step then consists of the update rule

(55) $${\left(\begin{array}{c} \mu \\ \Phi_{i}\\ H_{i} \end{array}\right)}^{k+1}={\left(\begin{array}{c} \mu \\ \Phi_{i}\\ H_{i} \end{array}\right)}^{k}+s_{k}\;\cdot \;\nabla J_{\ell }\left({\left(\mu ,\Phi_{i},H_{i}\right)}^{k}\right)\;.$$

**Figure 5:**
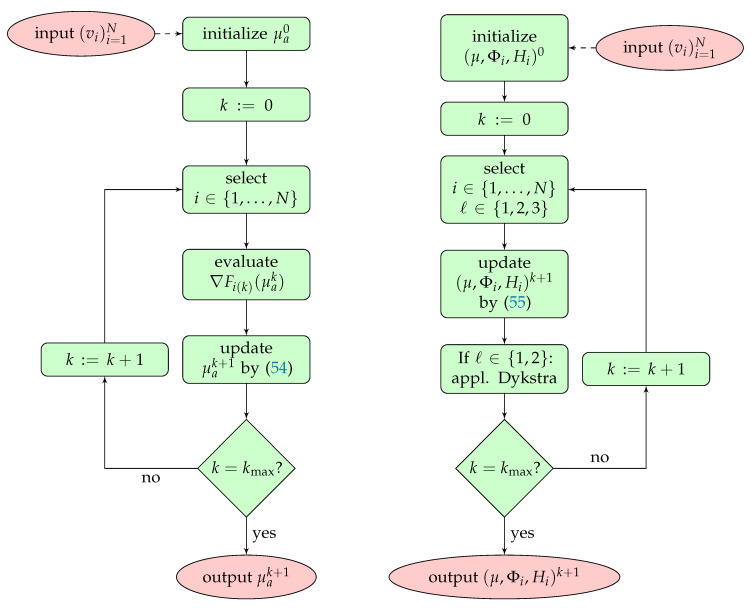
Flowcharts of stochastic gradient algorithms for QPAT proposed in this paper. **Left**: algorithm based on the standard formulation (19). **Right**: algorithm based on the novel MULL formulation (35). The update (54) using the standard formulation requires solving the forward RTE and the adjoint RTE, which is not required by (55) with the MULL formulation. Simulations are performed with N=4.

Dykstra’s algorithm for smoothing the μ component is applied after each iterative step when ℓ∈{1,2}. Iteration (55) contains a gradient step for the RTE. Since one gradient step is not enough to obtain an appropriate approximation to the solution of the transport equation, we apply iteration (55) 40 times whenever ℓ=1 is chosen. In this situation, we apply the Dykstra iteration in the μ component after these 40 iteration steps, whereas the positivity projection is done in every step. Flowcharts of the stochastic gradient algorithms (standard and MULL formulations) are shown in Figure 5. For the projected stochastic gradient method, regularization of μ is done by incorporating the regularization functional $J_{4}$ in the random choice of functionals; see (38). The positivity restriction is realized with the cut projection $P_{D}\left(\mu \right)=\max\left\{0,\mu \right\}$ applied after every iterative step.

**Figure 6:**
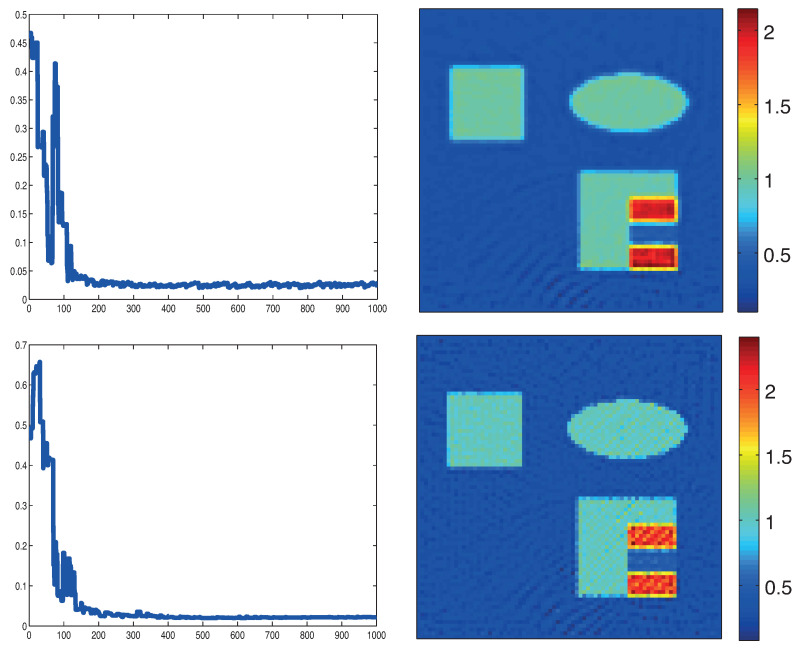
Reconstruction results based on the novel MULL formulation (35). **Top**: MULL-proximal stochastic gradient method based on the decomposition (38). **Bottom**: MULL-projected stochastic gradient method based on the decomposition (39). The left images show the relative reconstruction errors of the reconstructed absorption coefficient as a function of the number of iterations, whereas the right pictures show the results after the last iterations. (The phantom is as described in Figure 3.)

Figure 6 shows reconstructions with the stochastic gradient methods for the novel MULL formulation of QPAT (35). The results in the top row are for the MULL-proximal stochastic gradient algorithm with $\lambda =2\times 10^{-8}$ and in the the bottom row results for the MULL-projected stochastic gradient method with $\lambda =2\times 10^{-8}$ are shown. In both cases, we used 1000 iterations.

**Remark** **1.** Note that in the stochastic gradient methods for the novel MULL formulation of QPAT calculating the matrix vector product $M^{\left(h\right)}\;\cdot \;\Phi$ is the most costly part. In contrast, the standard formulation (19) requires the solution of the system $M^{\left(h\right)}c\left(h\right)=b^{\left(h\right)}$. Since the matrix $M^{\left(h\right)}$ is sparse only in its spatial domain, this is very time-consuming. On the other hand, the matrix $M^{\left(h\right)}$ (which is a discretization of $\theta \;\cdot \;\nabla_{x}+\mu_{a}+\mu_{s}\left(I-K\right)$) has a simple dependence on the variables $\mu_{a},\mu_{s}$. We therefore can compute the velocity entries of $M^{\left(h\right)}$ at the beginning of the iterative process to save computation time.

The reconstruction times for the final reconstructions using all methods described above are shown in Table 1. For the standard formulation of QPAT, the reconstruction times seem to be in accordance with reported results using gradient or Newton-type methods for QPAT (see, for example, [48].) The methods based on the new MULL formulation (35) (after 1000 iterations) are faster than the methods based on the standard formulation (19) of QPAT (after 10, respectively, 40 iterations). From the relative reconstruction errors shown in Figure 4 and Figure 6, one notices that, opposed to the methods based on (19), the methods using the MULL formulation could even be stopped much earlier while still obtaining a comparable reconstruction quality. We roughly estimate a speedup of a factor 10 using the novel MULL formulation instead of the standard formulation of QPAT.

**Figure 7:**
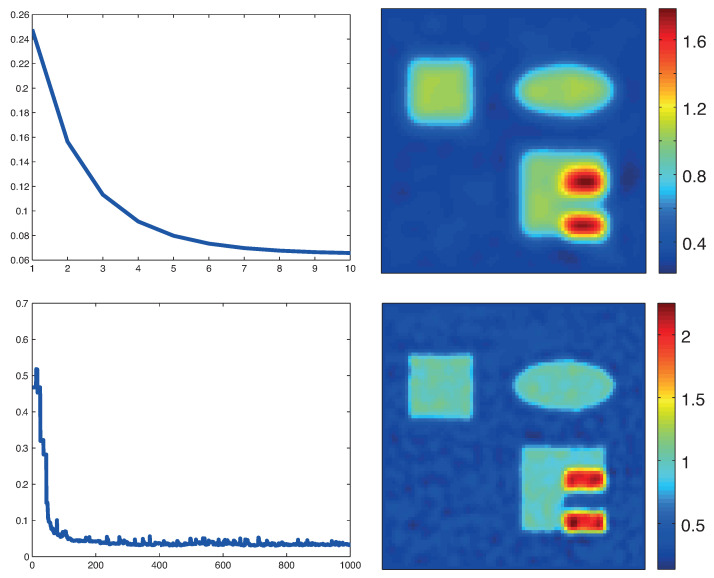
Reconstruction results from noisy data. **Top**: Proximal gradient method based on (19). **Bottom**: MULL-proximal stochastic gradient method. The left images show the relative reconstruction errors of the reconstructed absorption coefficient as a function of the number of iterations, whereas the right pictures show the results after the last iterations. (The phantom is as described in Figure 3.)

**Figure 8:**
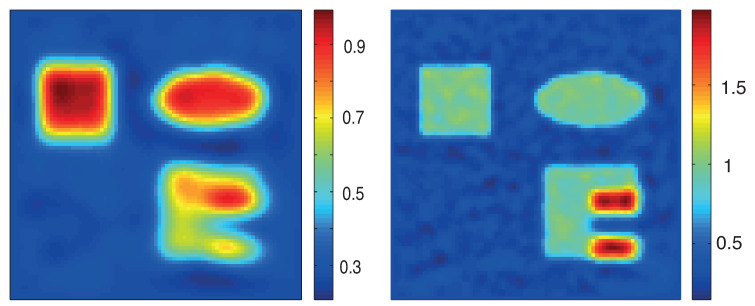
Reconstruction results from noisy data for two illuminations. **Left**: proximal gradient method based on (19) using 10 iterations. **Right**: MULL-proximal stochastic gradient method using only 500 iterations. The phantom is as described in Figure 3 and, for the reconstruction methods, we use two consecutive illuminations (from the top and from the left). The reconstruction time has been about 14 h for the method based on the standard formulation (19) and 3 h for the proposed MULL-proximal stochastic gradient method.

In Figure 7, we show results for noisy data using the proximal gradient method based on the standard formulation (19) (top) and the proximal stochastic gradient method using the MULL formulation for QPAT (bottom). The regularization parameter is chosen as in the exact data case. Finally, in Figure 8, we show reconstruction results using only two consecutive illuminations applied from the top and from the left with noisy data. We use 10 iterations for the proximal gradient algorithm based on (19) (shown in left image in Figure 8) and 500 iterations for the stochastic gradient algorithms based on the MULL formulation (35) (shown in the right image in Figure 8).

## 6. Conclusions

In this paper, we developed efficient proximal stochastic gradient methods for image reconstruction in multi-source QPAT. We used the RTE as an accurate model for light transport and employed the single stage approach for QPAT introduced in [18]. One class of the proximal stochastic gradient methods has been developed based on the standard formulation for QPAT given in (19). Additionally, we developed another class using proximal stochastic gradient methods for the new MULL formulations (35) and (36) for QPAT. Besides proposing proximal stochastic gradient methods for QPAT, we also consider the formulations (35) and (36) as the main contributions of the present article. These new formulations avoid the time-consuming evaluation of the RTE at each iteration and allow for treating the QPAT problem as a constrained optimization problem, which enables the use of a variety of numerical algorithms. Here, we used a penalty approach in combination with stochastic gradient methods for the solution. Future work will be done in the direction of developing new algorithms based on (35) and (36). Additionally, we will investigate the use of different regularization terms in (36). Finally, the theoretical convergence analysis of proximal gradient algorithms and other iterative algorithms for solving (35) will be the subject of future research.

## Figures and Tables

**Table 1:** Reconstruction times for all methods. Recall that one iteration of the proximal stochastic gradient method is approximately four times cheaper than one iteration of the full proximal gradient method (both based on (19)). Further recall that one step in the methods based on the MULL formulation (35) is much less time consuming than for the methods based on (19); see Remark 1.

Algorithm	Model	Update	No. Iterations	Reconstruction Time
Proximal gradient	(19)	(23)	10	27.2 h
Proximal stochastic gradient	(19)	(31)	30	24.4 h
MULL-proximal stochastic gradient	(35)	(49)	1000	14.7 h
MULL-projected stochastic gradient	(35)	(50)	1000	11.9 h
